# A Sea Anemone Tentacle-Inspired Capacitive 3D Force Flexible Tactile Sensor for Human–Machine Interaction and Encoding Communication Applications

**DOI:** 10.3390/polym18111388

**Published:** 2026-06-03

**Authors:** Xide Wang, Qingyan Fang, Shusong Li, Wuheng Xun, Ping Xin, Fanlong Liu, Bin Li, Rongwei Shi, Lupeng Lin

**Affiliations:** 1 School of Artificial Intelligence, Harbin University, Harbin 150086, China; wxd502@hrbu.edu.cn (X.W.); lishusong@hrbu.edu.cn (S.L.); pingxin507@163.com (P.X.); 17390614562@163.com (F.L.); 2 School of Information, Harbin Guangsha College, Harbin 150086, China; 3 School of Information Engineering, Dalian Ocean University, Dalian 116023, China; 4 School of Information Technology, Jilin Normal University, Siping 136000, Chinao19281928srw@163.com (R.S.)

**Keywords:** flexible tactile sensor, three-dimensional force, capacitive sensing, bioinspired structure, sea anemone mimic, wearable electronics, human–machine interaction

## Abstract

Sea anemones detect external stimuli through the deformation of their soft tentacles, which exhibit multi-directional force sensitivity. Inspired by this mechanism, we designed a capacitive three-dimensional force flexible tactile sensor composed of a hollow hemisphere and a hollow cylinder. The device was fabricated using 3D printing combined with a Layer-By-Layer assembly process. For normal forces, the sensor achieved sensitivities of approximately 0.66 N^−1^ in the 0–1 N range and 0.15 N^−1^ in the 2–10 N range. For tangential forces, the four symmetrically distributed electrodes exhibited opposite monotonic capacitance variation trends. The sensor exhibited a force resolution of 0.02 N, a lower detection limit of 0.04 N, a hysteresis error as low as 3.5%, and a response/recovery time of up to 50 ms under a 0–10 N load. Moreover, the device demonstrated good stability under 1000 load–unload cycles and over a temperature range from 20 °C to 100 °C. Its utility was further validated through multi-scenario applications, including game controller manipulation, gripper-based object recognition, Morse code and Huffman coding transmission, as well as multi-joint human motion detection. These results demonstrate that the proposed bioinspired sensor offers a promising solution for flexible force sensing, human–machine interaction, and wearable health monitoring.

## 1. Introduction

Flexible tactile sensors have attracted considerable attention for applications in wearable electronics, human–machine interaction, and intelligent robotics due to their ability to conform to curved surfaces and provide real-time mechanical feedback [1,2,3,4,5,6,7,8]. Among various transduction mechanisms, capacitive sensors offer distinct advantages including low power consumption, high signal-to-noise ratio, and excellent long-term stability [9,10,11]. However, most conventional capacitive sensors are limited to detecting only normal (vertical) forces, which is insufficient for tasks requiring multi-directional tactile perception such as dexterous robotic grasping and prosthetic control [12,13,14]. Several efforts have been made to develop three-dimensional (3D) force sensors. For example, some researchers have used carbon-based fillers in elastomeric matrices to achieve pressure sensitivity, but these materials often suffer from poor dispersion and inconsistent electrical performance [15,16,17,18,19,20,21,22]. Others have employed liquid metal or ionic gels as sensing layers, yet such approaches face challenges in long-term stability, leakage risks, or complex encapsulation [23,24,25]. In contrast, polydimethylsiloxane (PDMS) stands out as an ideal flexible substrate due to its excellent flexibility, chemical inertness, hydrophobicity, high resilience, thermal stability, fatigue resistance, electrical insulation, and good biocompatibility [26,27,28,29,30]. Meanwhile, conductive silver adhesive offers superior adhesion strength, charge transfer capability, thermal reliability, bendability, and aging resistance, making it highly suitable for reliable electrode fabrication in flexible sensors [31,32,33]. Despite these material advantages, relatively few studies have explored the combination of PDMS and conductive silver adhesive for constructing a simple, low-cost 3D force sensor that simultaneously integrates information encoding capabilities.

Herein, we present a sea anemone tentacle-inspired capacitive 3D force flexible tactile sensor that fully leverages the benefits of PDMS and conductive silver adhesive. The sensor features a hollow hemisphere and a hollow cylinder assembled structure with four symmetrically distributed top electrodes (made of conductive silver adhesive) on the PDMS substrate and a bottom common electrode, using an air gap as the dielectric layer. PDMS serves as both the flexible supporting substrate and the adhesive encapsulation material, while the conductive silver adhesive ensures robust electrical interconnection and stable signal output. The device is fabricated via a simple combination of 3D printing and layer-by-layer assembly, offering low cost and high reproducibility. We systematically evaluate key performance parameters including sensitivity, force resolution, detection limit, hysteresis, response/recovery time, temperature stability, and mechanical durability, and demonstrate practical applications in game controller joystick sensing, robotic hand grasping with PCA-based object recognition, multi-joint human motion monitoring (wrist, finger, neck, elbow, knee, and foot), and encoding communication using Morse code and Huffman coding.

In recent years, several bioinspired capacitive tactile sensors have been reported in Refs. [34,35,36,37,38,39,40], including river-valley-inspired [34], frog-leg-inspired [35], cheetah-leg-inspired [36], inverted-pyramid-structured [37], grey-kangaroo-leg-inspired [38], ionogel-electrode-based [39], and inverted-truncated-cone-structured designs [40]. Although these configurations each offer advantages in sensitivity or detection range, most require photolithography, complex molds, or specialized materials (e.g., ionogels), and suffer from issues related to long-term stability or fabrication complexity. More importantly, none of the above works have demonstrated information encoding (e.g., Morse code, Huffman coding) as a functional application of the sensor.

In contrast, the innovations of our sensor lie in: (i) a unique geometry combining hollow hemispheres and hollow cylinders, enabling decoupled responses to normal and tangential forces using only four symmetric electrodes; (ii) a simple fabrication process requiring only 3D printing and layer-by-layer assembly, without the need for photolithography, thus offering low cost; and (iii) being the first demonstration of coding communication functionality based on Morse code and the Huffman algorithm on a flexible 3D force tactile sensor. Our results confirm that the synergistic use of PDMS and conductive silver adhesive enables a high-performance 3D force sensor with excellent flexibility, durability, and multifunctional capabilities, outperforming previously reported devices that rely on less compatible or less stable material systems.

## 2. Experimental

The three main raw materials used in this experiment are polydimethylsiloxane (PDMS, trade name Sylgard 184) from Dow Corning (Midland, TX, USA), an organosilicon conductive silver adhesive (grade LX-50) from Nanjing Chuangyiyou Electronic Technology Co., Ltd. (Nanjing, China), and silicone rubber (grade GD401) from Zhonghao Chenguang Chemical Research Institute Co., Ltd. (Zigong, China). Both PDMS and silicone rubber exhibit excellent flexibility, chemical inertness, hydrophobicity, high resilience, thermal stability, fatigue resistance, electrical insulating properties, and good biocompatibility. These characteristics make them particularly suitable as flexible supporting substrates and adhesive encapsulation materials. The conductive silver adhesive, owing to its superior adhesion strength, charge transfer capability, thermal reliability, bendability, and aging resistance, has been widely used for conductive interconnection of sensor electrodes, thereby helping to enhance the device’s sensing accuracy and signal response speed.

**Figure 1:**
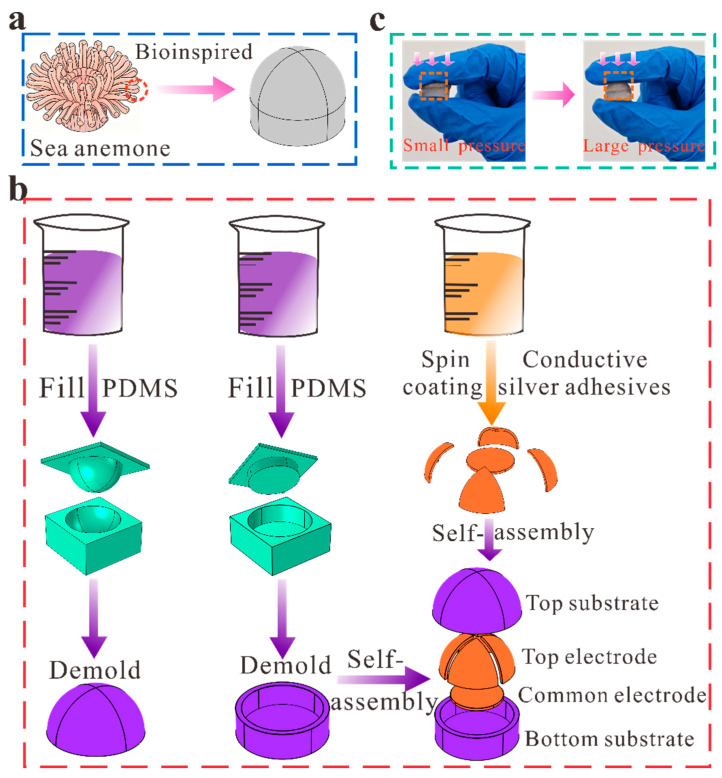
Sea anemone tentacle-inspired capacitive three-dimensional force flexible tactile sensor: (**a**) Schematic diagram of the structural design; (**b**) Fabrication and assembly process; (**c**) Deformation comparison under low pressure and high pressure.

In this work, a capacitive three-dimensional force flexible tactile sensor inspired by the morphology of sea anemone tentacles is designed. Its structural schematic is shown in Figure 1a. The fabrication of the device integrates 3D printing and Layer-By-Layer assembly processes, resulting in a simple and easily achievable overall procedure. At the initial stage of fabrication, reverse modeling of each component is carried out using the COMSOL Multiphysics simulation platform to determine the geometric parameters of the required molds. The STL files of the molds are imported into the Cura slicing software v15.02.1 for processing and then transferred to an A6 3D printer (with a forming accuracy of 0.1 mm) manufactured by Shenzhen Aurora Technology Co., Ltd. (Shenzhen, China), using polylactic acid (PLA) filament to complete the additive manufacturing of the molds.

After the mold printing process is finished, Sylgard 184 base and curing agent were mixed at a weight ratio of 10:1, and the mixture was stirred using a magnetic stirrer (model NB-1S, Suzhou Jiulian Technology Co., Ltd. (Suzhou, China)) at 50 rpm for 30 min at room temperature until bubbles disappeared, after which the bubble-free mixture was poured into the molds. The PDMS was then cured in an oven at 50 °C for 12 h and subsequently cooled naturally to room temperature. PDMS is poured into the molds. The bubbles generated during pouring are eliminated by standing for one hour. The molds are then placed into a YLE-2000 vacuum oven (produced by Lichen Technology Co., Ltd. (Changsha, China)) and kept at 50 °C for approximately twelve hours to allow the PDMS to fully cure. For electrode fabrication, the conductive silver adhesive (LX-50) was mixed with the curing agent at a weight ratio of 10:1, and then uniformly applied onto the electrode surfaces using a small scraper with a coating thickness of approximately 0.5 mm, followed by curing at room temperature for 24 h until fully hardened.

Then, one end of each connecting wire is introduced into the substrate at the positions of the bottom common electrode and the four top electrodes, respectively, while the other ends are led out of the substrate to test their electrical conductivity. Finally, silicone rubber is used as the bonding medium to perform LBL assembly of all components, followed by another drying step in a vacuum environment at 50 °C for six hours to ensure firm interlayer connections. At this point, the sensor fabrication is completed. Detailed preparation steps and assembly schematics are shown in Figure 1b.

Regarding the structural design, the sensor consists of a hollow hemisphere (radius 8 mm) and a hollow cylinder (bottom radius 8 mm, height 5 mm) joined together. The PDMS wall thicknesses of the hemisphere and the cylinder are both 1.5 mm. Four top electrodes are symmetrically distributed on the inner surface of the hemispherical shell, while the common electrode covers the bottom area of the cylinder. Due to the curved mating surface between the hemisphere and the cylinder, the initial air-gap thickness between the top electrodes and the bottom common electrode varies with position, ranging from approximately 3.5 mm (minimum) to 10 mm (maximum), with a typical average value of about 5–6 mm in the working region. The four top electrodes are sector-shaped, obtained by equally dividing the hemispherical inner surface into four parts, each with a theoretical area of 59.87 mm^2^ and a gap of 0.5 mm between adjacent electrodes. The bottom common electrode is circular with a diameter of 10 mm, corresponding to an area of 78.54 mm^2^. The conductive silver adhesive used for the electrodes dries to a thin layer with a thickness of less than 0.5 mm (estimated). Five flexible wires are used as leads: four are embedded into the four top electrode regions, and one is embedded into the bottom common electrode. All leads are fixed with conductive silver adhesive and sealed with silicone rubber. An air gap serves as the dielectric layer of the device, thus forming a three-dimensional force sensor.

The results shown in Figure 1c indicate that, whether under low or high pressure, this three-dimensional force flexible sensor exhibits significant compressive deformation capacity and elastic recovery characteristics, demonstrating its great potential for applications in the field of flexible sensing.

**Figure 2:**
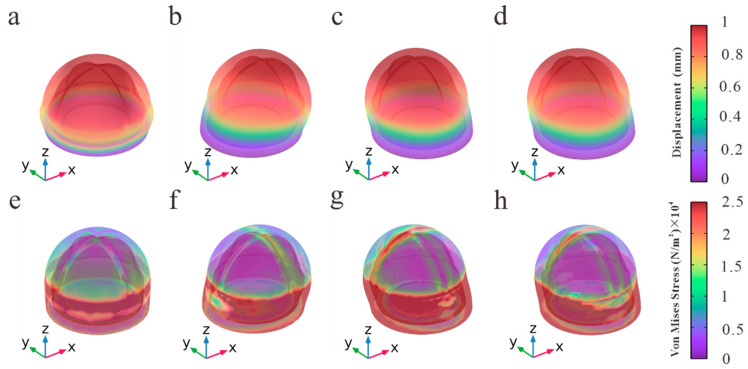
Finite element simulation results under 1 N: (**a**) Displacement (negative *z*-axis); (**b**) Displacement (positive *x*-axis); (**c**) Displacement (positive *y*-axis & negative *z*-axis); (**d**) Displacement (positive *y*-axis); (**e**) Stress (negative *z*-axis); (**f**) Stress (positive *x*-axis); (**g**) Stress (positive *y*-axis & negative *z*-axis); (**h**) Stress (positive *y*-axis).

To theoretically validate the structural design and understand its mechanical response under various loading conditions, finite element analysis was performed using COMSOL Multiphysics 6.2. Figure 2a–d present the displacement response simulation results of the finite element model under different loading conditions, all with a load magnitude of 1 N. To systematically evaluate the deformation behavior of the structure under multi-directional external forces, four typical loading conditions were set: a unidirectional load along the negative *z*-axis, a unidirectional load along the positive *x*-axis, a combined load consisting of the positive *y*-axis and negative *z*-axis, and a unidirectional load along the positive *y*-axis. Comparative analysis of the displacement distribution contours under different loading conditions intuitively characterizes the deformation amplitude, deformation trend, and main response regions of the model under external forces in various directions.

Figure 2e–h present the stress distribution results obtained under the same simulation environment and boundary conditions, also with a load magnitude of 1 N. The loading conditions remain consistent with those in the displacement simulation, corresponding to the negative *z*-axis direction, positive *x*-axis direction, combined positive *y*-axis and negative *z*-axis direction, and positive *y*-axis direction, respectively. Analysis of the stress concentration regions and stress distribution patterns under different loading conditions further reveals the mechanical response characteristics of the structure under multi-axis loading, providing theoretical support and simulation-based guidance for structural design, sensitive region determination, and subsequent performance optimization of flexible tactile sensors.

To evaluate the capacitance variation in the sensor, a capacitance information acquisition system was established in this study. The core components of this system include a TH2832 LCR digital bridge manufactured by Tonghui Electronics (Changzhou, China) and an STM32VET6 control microprocessor supplied by STMicroelectronics (Geneva, Switzerland). To simulate the force state under real working conditions, a ZQ990B universal testing machine produced by Weisi Precision Instruments Co., Ltd. (Dongguan, China) was used during the evaluation, with a force measurement accuracy of 0.01 N. For temperature characteristic testing, the sensor was placed on an LC-EH45 heating platform from Lichen Instrument (Shanghai, China) to achieve data measurement under different temperature environments.

It is hereby declared that all human subjects involved in this study gave their informed consent to participate in the experiments. Regarding the demonstration experiments involving wearing the pressure sensor, explicit authorization was obtained from the volunteers.

## 3. Results and Discussion

Sea anemones are widely distributed in intertidal zones and shallow sea areas, relying on numerous soft tentacles to detect the surrounding environment. These tentacles produce sensitive deformation responses when subjected to water flow impact or touching from any direction, helping the sea anemone capture prey or avoid threats. Inspired by this multi-directional force perception mechanism, we designed a capacitive three-dimensional force flexible tactile sensor assembled from a hollow hemisphere and a hollow cylinder.

**Figure 3:**
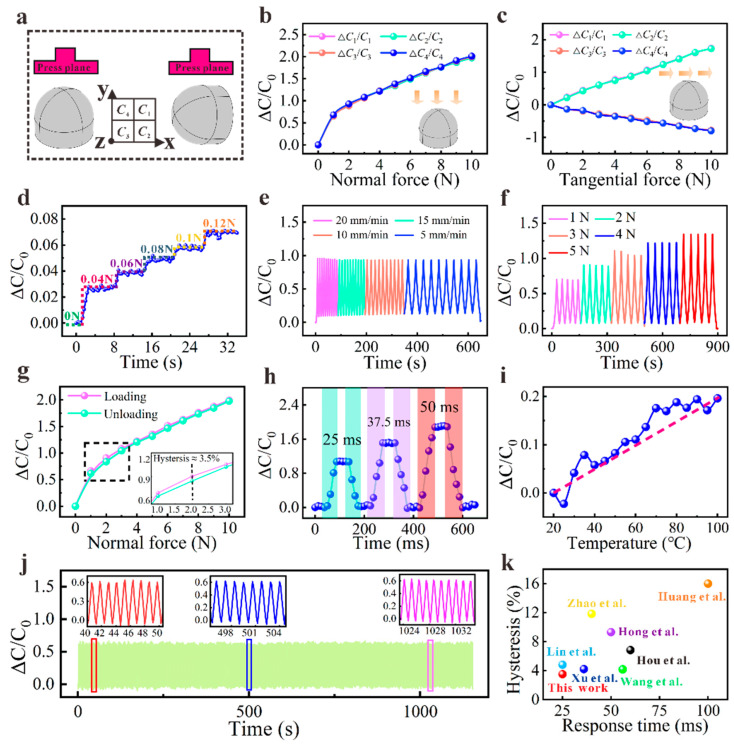
Performance evaluation of the sensor. (**a**) Schematic of the experimental test setup and electrode layout. (**b**) Capacitance change in the sensor under normal forces of 0–10 N. (**c**) Dynamic capacitance response of the sensor under tangential forces of 0–10 N. (**d**) Force resolution and minimum detection limit of the device. (**e**) Capacitance output of the sensor under variable loading rates (5, 10, 15, 20 mm/min). (**f**) Response characteristics of the sensor under step normal forces (1–5 N). (**g**) Hysteresis characteristic test. (**h**) Response and recovery time measurement. (**i**) Effect of temperature fluctuation on device performance. (**j**) Durability performance of the sensor under 1000 cyclic tests at 1 N. (**k**) Comparison of response time and hysteresis error with similar studies [34,35,36,37,38,39,40].

When evaluating the sensor performance, the leads were first connected to the measurement channels of the LCR meter, and then the device was fixed on the horizontal platform of a standard test setup. Normal force tests were performed by loading vertically. Since the setup can only apply pressure through vertical movement, tangential force tests were carried out by placing the sensor sideways on the horizontal platform. The corresponding test configuration and electrode distribution are shown in Figure 3a. According to the electrode arrangement orientation shown in the figure, under the action of tangential force along the *x*-axis, *C*_1_ and *C*_2_ exhibit the same capacitance variation trend, with capacitance values increasing monotonically as the tangential force increases. In contrast, the capacitance changes in *C*_3_ and *C*_4_ also remain synchronized, but their capacitance values decrease monotonically with increasing tangential force. This evaluation aims to comprehensively investigate the key performance metrics of the sensor, including sensitivity, hysteresis, dynamic response, force resolution, repeatability, and temperature characteristics.

Sensitivity, as one of the key static performance metrics for evaluating a sensor, describes the ratio between the output change in the device and the input change. This parameter can also be obtained from the slope of the curve shown in Figure 3b, with the specific calculation formula: *S* = ((*C* − *C*_0_)/*C*_0_)/*F*, where *C* represents the real-time capacitance of the sensor under given input conditions, *C*_0_ is its initial capacitance, and *F* corresponds to the magnitude of the applied external load. When a normal force is applied to the sensor, the responses of the four sets of capacitors are shown in Figure 3b. In the force range of 0 to 1 N, the sensitivity of the device is approximately 0.66 N^−1^; when the load increases to the range of 2 to 10 N, the sensitivity is approximately 0.15 N^−1^. Figure 3c shows the dynamic capacitance response curves of the four electrodes during the tangential force loading along the *x*-axis direction. It can be clearly observed that as the tangential force gradually increases, the capacitance signals of *C*_1_ and *C*_2_ change synchronously, both showing a monotonically increasing trend. In contrast, *C*_3_ and *C*_4_ also maintain a consistent variation step, but their capacitance values decrease monotonically. The change directions of these two groups of capacitors are exactly opposite. The detailed mechanism of capacitance variation for each capacitor has been elaborated in the previous discussion and will not be repeated here.

Force resolution characterizes the minimum external force change that a sensor can perceive, while the detection limit reflects the lowest force level that can be accurately measured. Both of these metrics are important criteria for evaluating the overall performance of a sensor. The sensor developed in this study exhibits excellent characteristics in both of the above parameters, possessing both a low resolution threshold and a low detection limit. As shown in Figure 3d, the force resolution of the device reaches 0.02 N, and the detection limit is as low as 0.04 N. This means that the sensor can not only accurately capture weak force variation signals but also detect extremely small force loads. With such outstanding sensing capability, the device shows broad application prospects in the field of high-precision measurement.

In practical working conditions, evaluating the sensor’s response to different loading rates under a constant force, as well as its feedback to different force magnitudes at a fixed loading rate, is a key step in assessing its performance. To this end, we tested the capacitance output of the device under various loading rates at a normal force of 2 N. As shown in Figure 3e, the sensor exhibits good response characteristics at loading rates of 5 mm/min, 10 mm/min, 15 mm/min, and 20 mm/min. To further verify its performance, we kept the loading rate constant at 10 mm/min and applied normal forces of 1 N, 2 N, 3 N, 4 N, and 5 N sequentially. The results in Figure 3f show that the sensor maintains a stable response output under different load conditions. This fully demonstrates that the device maintains excellent performance under diverse loading conditions.

Hysteresis is another key factor in evaluating sensor performance. It describes the inconsistency of the output response during forward loading and reverse unloading under the same input conditions. Figure 3g shows the hysteresis characteristics of the device. The measured hysteresis error is only 3.5%. This result indicates that the sensor provides highly consistent output signals when performing repetitive measurement tasks. A low hysteresis error is crucial for ensuring the measurement accuracy and reliability of the device in various application scenarios.

Response/recovery time, which refers to the time interval from when the sensor receives an input stimulus to when it generates an output response, is also an important parameter for evaluating its dynamic performance. As shown in Figure 3h, the sensor exhibits extremely fast response speed: under the 0–2 N range, the response/recovery is 25 ms; under the 0–5 N range, it is 37.5 ms; and under the 0–10 N range, it is 50 ms. This fast response capability may originate from its rational structural arrangement and optimized material system, enabling the device to respond rapidly upon sensing a force signal and quickly return to its initial state after the stimulus is removed. Such fast response and recovery characteristics make it competent for high-frequency dynamic measurement tasks, providing accurate real-time feedback even in rapidly changing environments.

In practical applications, evaluating the influence of temperature on sensor performance is essential. As the ambient temperature increases, the air dielectric layer and the PDMS substrate inside the device undergo thermal expansion, causing the distance between the top electrodes and the common electrode to decrease slightly, and thus the relative capacitance change curve rises moderately. To this end, we recorded the capacitance variation in the sensor by increasing the temperature in steps of 5 °C from 20 °C to 100 °C; the results are shown in Figure 3i.

Experiments show that temperature fluctuations have only a very limited effect on sensor performance, which provides an important guarantee for ensuring stable operation of the device under different environmental conditions.

To further evaluate the performance of the sensor under low-temperature conditions, we conducted additional tests in the range of −20 °C to 20 °C. The sensor was placed in a household refrigerator, and the capacitance response was measured during the natural cooling process after power-off. The specific procedure was as follows: (1) The sensor was placed in the refrigerator compartment, and the capacitance was recorded every ~5 °C during cooling from room temperature (20 °C) to 0 °C. (2) The sensor was then transferred to the freezer compartment, and the capacitance was recorded every ~5 °C during cooling from 0 °C to −20 °C. The experimental results show that the capacitance variation in the sensor is relatively small in the range of −20 °C to 20 °C, demonstrating good low-temperature stability.

To verify the reliability of the sensor during long-term operation, mechanical durability testing is an indispensable step. Figure 3j shows the results of the device after 1000 cyclic tests under a load of 1 N. The test data demonstrate that the sensor maintained good stability throughout the entire cycle, with no significant performance degradation or signal drift. The findings of this test fully confirm the excellent characteristics of the sensor in terms of stability, long-term reliability, and consistency.

An additional 1000-cycle durability test was performed under 10 N (see Supplementary Figure S1). Due to the relatively high applied load, a gradual increase in capacitance was observed during the test; however, the sensor remained functional throughout. This gradual drift is attributed to stress relaxation and residual deformation of PDMS under repeated high loads. A low-load cyclic test was also conducted under 0.2 N for 5000 cycles (see Supplementary Figure S2). Although a slight baseline drift was observed, the sensor remained functional throughout.

In Figure 3k, we conducted a comprehensive comparative analysis of the designed three-dimensional force sensor against similar recent studies [34,35,36,37,38,39,40] in terms of response time and hysteresis error. The comparison results show that the sensor developed in this study exhibits obvious advantages in both response speed and hysteresis performance. Its fast response characteristic is of critical importance for real-time monitoring and rapid feedback applications, while the low hysteresis error effectively ensures high consistency of the output signal during repeated loading and unloading processes, which is particularly crucial for scenarios requiring high repeatability and high measurement reliability.

To facilitate an objective performance comparison between our sensor and recent related works [34,35,36,37,38,39,40], the key parameters are summarized in Table S1 in the Supplementary Materials. As shown in the table, our sensor exhibits comparable overall performance while demonstrating distinctive features in application expansion, such as encoding communication.

**Figure 4:**
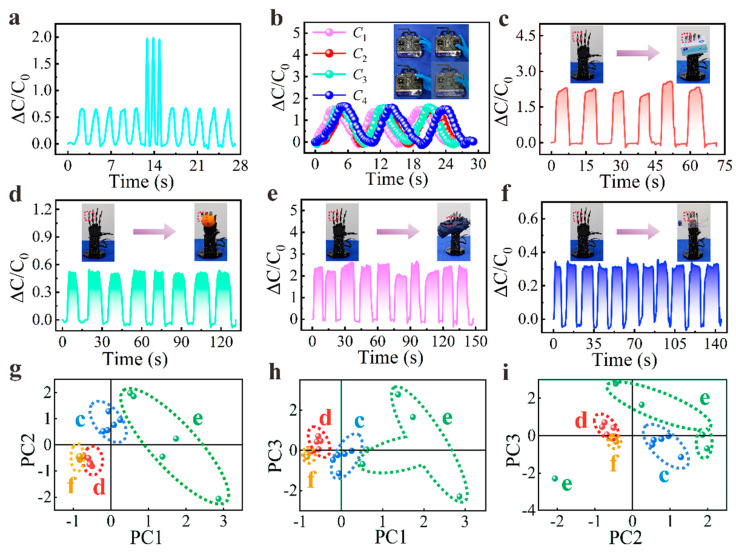
Application of the sensor in force feedback and grasping. (**a**) Robustness verification of the sensor under pressure impact testing. (**b**) Multi-directional force sensing attached to a game controller joystick. (**c**–**f**) Experiments of a robotic hand grasping different objects (tissue, orange, umbrella, empty bottle). (**g**–**i**) PCA-based clustering analysis of grasping signals.

Robustness is an important indicator of whether a device can maintain reliable operation under complex, variable, or even harsh conditions. To verify the stability of our sensor, we conducted a pressure impact test, as illustrated in Figure 4a. The test results show that after a sudden increase in pressure, the output performance of the sensor remained consistent with its state before the pressure increase, without significant fluctuation or performance degradation. This phenomenon fully demonstrates that the device possesses excellent robustness.

As shown in Figure 4b, after attaching the sensor to the joystick of a game controller, it is able to perceive forces from different directions and of varying magnitudes in real time. As the applied force gradually increases, the capacitance values of the four capacitors rise synchronously, exhibiting a good force–capacitance response relationship. The differences among the capacitance output signals mainly arise from changes in the joystick’s tilt angle, force direction, and rotation speed.

Figure 4c–f present the experimental results of grasping tests performed on different objects after integrating the sensor into a robotic hand. The grasped objects include a tissue, an orange, an umbrella, and an empty bottle. During the grasping process, the sensor generates response signals upon contact pressure, and the results show stable output with good repeatability.

Figure 4g–i further present the clustering results of the grasping signals based on principal component analysis (PCA). Through feature extraction and dimensionality reduction, the experiment successfully classified the grasping actions of the same object into one cluster, confirming that the robotic hand equipped with the sensor can effectively distinguish the grasping behaviors of most different objects. In Figure 4i, the cumulative variance contribution rates of PCA2 and PCA3 are relatively small, carrying very limited effective feature information; therefore, the issue of insufficient capture of the umbrella’s center-of-gravity shift and geometric anisotropy on this plane has little impact on the overall differentiation result.

**Figure 5:**
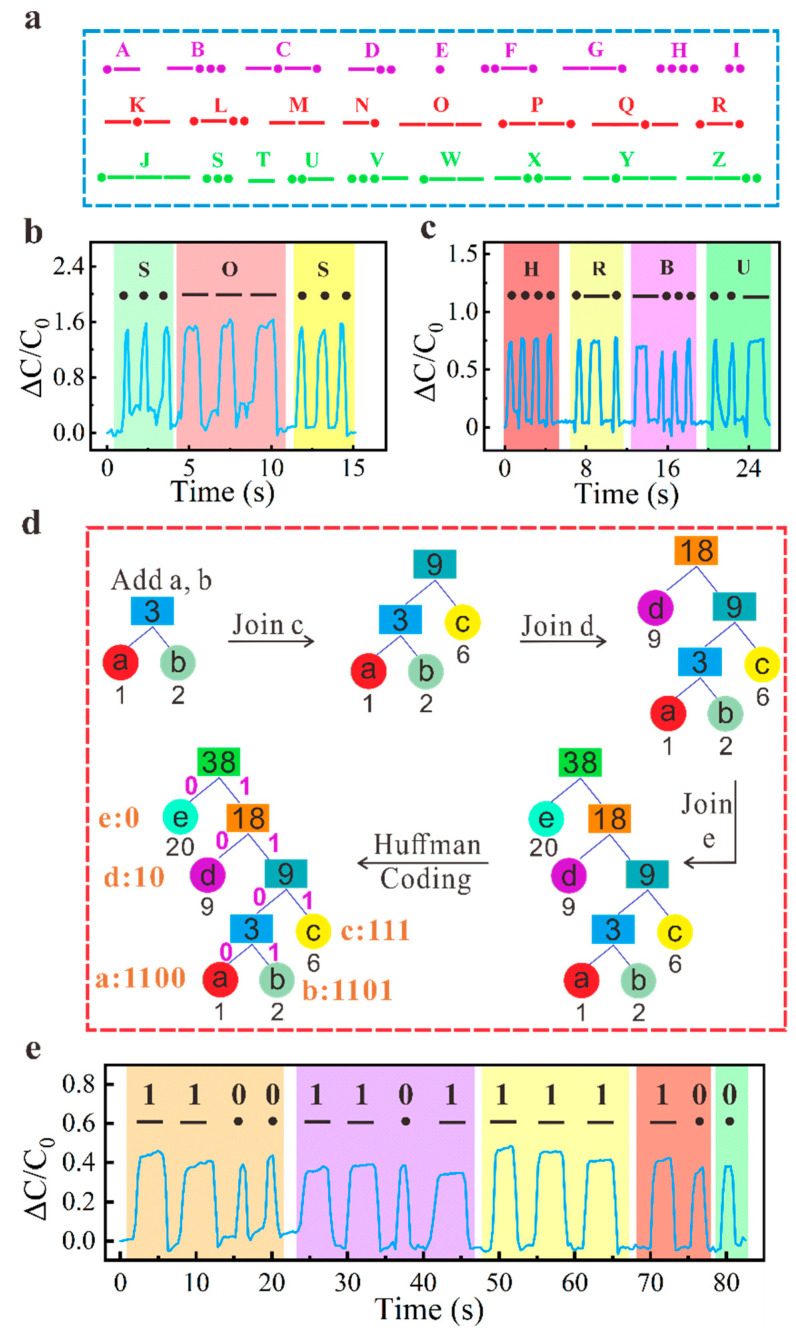
Application of the sensor in encoded communication. (**a**) Morse code table corresponding to 26 letters. (**b**,**c**) Output demonstration of Morse code “SOS” and “HRBU”. (**d**) Huffman tree and code construction for the weight set {a = 1, b = 2, c = 6, d = 9, e = 20}. (**e**) Binary sequence of Huffman code output by the sensor.

Morse code encodes letters as dot–dash sequences. Using the sensor, volunteers tapped short presses (dots) or long presses (dashes). Figure 5a shows the Morse code table corresponding to the 26 English letters. Figure 5b,c show the sensor outputs for ‘SOS’ and ‘HRBU’. We further implemented Huffman coding, an optimal prefix-free encoding scheme. Using the weight set {a = 1, b = 2, c = 6, d = 9, e = 20}, we constructed a Huffman tree (Figure 5d). The sensor’s short/long presses were mapped to binary 0/1, generating the encoded sequence shown in Figure 5e.

The above applications not only improve the transmission efficiency of information encoding but can also be extended to various scenarios requiring compact coding, demonstrating new application potential for the sensor in data compression and encoded communication.

**Figure 6:**
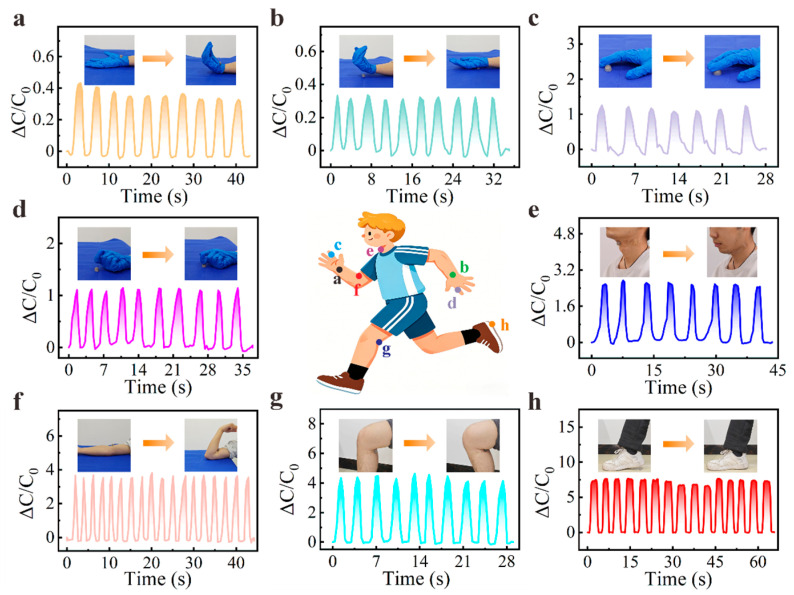
Wearable human motion monitoring demonstration. (**a**) Wrist forward-bending response. (**b**) Wrist backward-bending response. (**c**) Finger joint forward-pressing test. (**d**) Finger joint reverse-pressing test. (**e**) Neck-bending signal output. (**f**) Elbow pit-bending response. (**g**) Popliteal fossa-bending response. (**h**) Plantar pressure sensing.

To verify the practical performance of the sensor in human motion monitoring, we conducted a series of wearable demonstration experiments, and the results are shown in the corresponding figures. Figure 6a,b present the capacitance signals generated by wrist bending when the device was attached to the front and back of a volunteer’s wrist, respectively, showing stable and reliable output. Figure 6c,d record the sensor’s responses during forward pressing and reverse pressing of a finger joint, respectively, where the output waveforms of each set of actions maintained good consistency. Figure 6e shows the capacitance change caused by neck bending after fixing the device on the neck, with equally stable signal output. Figure 6f,g present the sensor’s signal responses during bending of the elbow pit and popliteal fossa, respectively, and the waveform characteristics remained stable. Figure 6h demonstrates an application scenario where the sensor was used to detect plantar pressure during simulated human walking, with the capacitance signal showing stable variations.

During the experiments, the fluctuations in the capacitance signals may have arisen from the difficulty of the subjects in precisely controlling the force and rhythm while operating the pressure application device. Nevertheless, the sensor was still able to accurately capture these dynamic changes, verifying that the device can precisely detect pressure variations caused by movements of various parts of the human body, thus demonstrating good potential for wearable monitoring.

The human-subject tests involved three participants, all of whom are co-authors of this paper. The sensor was attached to the test site or platform using transparent adhesive tape. The number of repetitions for each movement equals the number of waveform cycles displayed in the corresponding figures. Owing to the non-invasive and low-risk nature of the experiments and the fact that only the authors served as subjects, formal approval from an Institutional Review Board (IRB) was not required by our institutional guidelines. Nonetheless, informed consent was obtained from all participating authors.

## 4. Conclusions

Inspired by the multi-directional force perception characteristic of sea anemone tentacles, this paper designs and fabricates a novel capacitive three-dimensional force flexible tactile sensor. The device adopts a composite structure of a hollow hemisphere and a hollow cylinder, combined with 3D printing and Layer-By-Layer assembly processes, achieving three-dimensional sensitivity detection, fast response, and low hysteresis. The application potential of the sensor was further demonstrated in several different use cases: multi-directional force sensing on a game controller joystick, gripper-based grasp-and-identification of different objects with principal component analysis-based signal clustering, information encoding and wireless transmission using both Morse code and Huffman coding, and multi-joint wearable human motion detection on the wrist, finger, neck, elbow, knee and sole of the foot. These results suggest that the proposed three-dimensional force flexible tactile sensor opens up a viable technical pathway and promising prospects in the fields of flexible electronics, intelligent robotics and personal healthcare monitoring. Future work will focus on further miniaturization of the device architecture, integration of multichannel signal acquisition circuits, and validation of long-term system reliability under real-world operating conditions.

## Data Availability

No new data were created or analyzed in this study.

## Supplementary Materials

The following supporting information can be downloaded at: https://www.mdpi.com/article/10.3390/polym18111388/s1. Figure S1. Durability test of the sensor under 10 N load for 1000 cycles. Figure S2. Cyclic test of the sensor under 0.2 N load for 5000 cycles. Table S1. Comparison of key performance parameters between our sensor and recent representative works [34,35,36,37,38,39,40].
