# Supplementary material for: A Sea Anemone Tentacle-Inspired Capacitive 3D Force Flexible Tactile Sensor for Human–Machine Interaction and Encoding Communication Applications
Source: Polymers (Basel). 2026 Jun 3;18(11):1388. doi: 10.3390/polym18111388 (PMC13259269; doi:10.3390/polym18111388)
Supplement: Supplementary file 1 [file polymers-18-01388-s001.zip › polymers-4311451-supplementary.pdf]

Table S1: Comparison of Key Performance Parameters of This Sensor with Recent Similar Works [34–40].

Force resolution	Maximum sensitivity	Response/Recovery time	Hysteresis	Reference
0.1 N	0.487 N^{-1}	36 /36ms	4.2 %	Ref. [34]
0.5 Pa	0.583kPa^{-1}	40/45 ms	11.84 %	Ref. [35]
9 Pa	0.74 kPa^{-1}	50/60 ms	9.3 %	Ref. [36]
0.02 N	0.551 N^{-1}	56/30 ms	4.17 %	Ref. [37]
13 Pa	1.202 kPa^{-1}	60/85 ms	6.83 %	Ref. [38]
——	0.51 kPa^{-1}	100/200 ms	16 %	Ref. [39]
0.02 N	0.2 N^{-1}	25/25 ms	4.8 %	Ref. [40]
0.02 N	0.679 N^{-1}	25/25 ms	3.9 %	This work

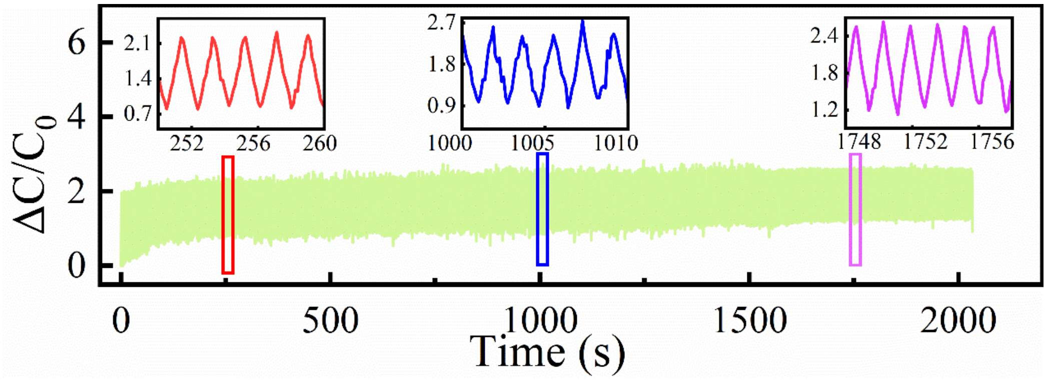

Figure S1. Durability test of the sensor under 10 N load for 1000 cycles.

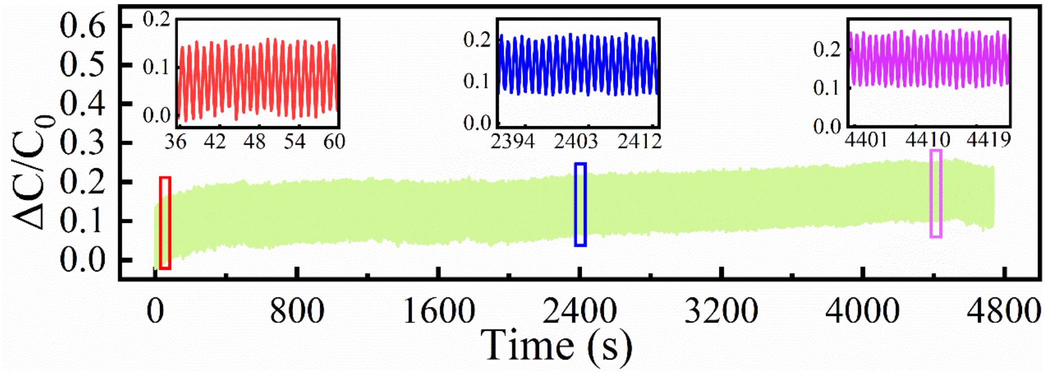

Figure S2. Cyclic test of the sensor under 0.2 N load for 5000 cycles.
